# Frequency of pathogenic germline variants in pediatric medulloblastoma survivors

**DOI:** 10.3389/fonc.2024.1441958

**Published:** 2024-08-09

**Authors:** Donald Rees, D. Matthew Gianferante, Jung Kim, Theodora Stavrou, Gregory Reaman, Yadav Sapkota, M. Monica Gramatges, Lindsay M. Morton, Melissa M. Hudson, Gregory T. Armstrong, Neal D. Freedman, Wen-Yi Huang, W. Ryan Diver, Adriana Lori, Wen Luo, Belynda D. Hicks, Jia Liu, Amy A. Hutchinson, Alisa M. Goldstein, Lisa Mirabello

**Affiliations:** ^1^ Division of Cancer Epidemiology and Genetics, National Cancer Institute, National Institutes of Health (NIH), Rockville, MD, United States; ^2^ Department of Pediatric Hematology and Oncology, Walter Reed National Military Medical Center, Bethesda, MD, United States; ^3^ Department of Pediatrics, Uniformed Services University of the Health Sciences, Bethesda, MD, United States; ^4^ Department of Public Health, Ministry of Health, Athens, Greece; ^5^ Division Cancer Treatment and Diagnosis, National Cancer Institute, National Institutes of Health (NIH), Bethesda, MD, United States; ^6^ Departments of Oncology and Epidemiology and Cancer Control, St. Jude Children’s Research Hospital, Memphis, TN, United States; ^7^ Division of Hematology and Oncology, Department of Pediatrics, Texas Children’s Hospital, Baylor College of Medicine, Houston, TX, United States; ^8^ Department of Population Science, American Cancer Society, Atlanta, GA, United States; ^9^ Cancer Genomics Research Laboratory, Frederick National Laboratory for Cancer Research, Frederick, MD, United States

**Keywords:** pathogenic, germline, pediatric, medulloblastoma, survivor

## Abstract

**Background:**

Medulloblastoma is the most common malignant brain tumor in children. Most cases are sporadic, but well characterized germline alterations in *APC*, *ELP1*, *GPR161*, *PTCH1*, *SUFU*, and *TP53* predispose to medulloblastoma. However, knowledge about pathogenic/likely pathogenic (P/LP) variants that predispose to medulloblastoma vary based on genes evaluated, patient demographics, and pathogenicity definitions.

**Methods:**

Germline exome sequencing was conducted on 160 childhood survivors of medulloblastoma. Analyses focused on rare variants in 239 known cancer susceptibility genes (CSGs). P/LP variants were identified using ClinVar and InterVar. Variants of unknown significance in known medulloblastoma predisposing genes (*APC*, *ELP1*, *GPR161*, *PTCH1*, *SUFU*, *TP53)* were further classified for loss of function variants. We compared the frequency of P/LP variants in cases to that in 1,259 cancer-free adult controls.

**Results:**

Twenty cases (12.5%) had a P/LP variant in an autosomal dominant CSG versus 5% in controls (p=1.0 x10^-3^), and 10 (6.3%) of these were P/LP variants in a known medulloblastoma gene, significantly greater than 0.2% observed in controls (p=1.4x10^-8^). The CSGs with the most P/LP variants in cases, and significantly higher than controls, were *ELP1 (*p=3.0x10^-4^) and *SUFU* (p=1.4x10^-3^).

**Conclusion:**

Approximately one in eight pediatric medulloblastoma survivors had an autosomal dominant P/LP CSG variant. We confirm several known associated genes and identify novel genes that may be important in medulloblastoma.

## Introduction

Medulloblastoma is the most common malignant brain tumor in children, with an incidence of up to 11 cases per 1,000,000 people worldwide (1–3). It is of embryonal origin and can be further classified into four molecular subgroups (WNT, Sonic Hedgehog (SHH), Group 3, and Group 4) based on distinctive tumor transcriptional and epigenetic signatures that demarcate clinically relevant subtypes (4, 5). Germline predisposing variants are important in the etiology of medulloblastoma. Although most cases are sporadic, medulloblastoma can occur in nevoid basal cell carcinoma (NBCCS) and Li-Fraumeni syndromes. These cancer predisposition syndromes are predominantly associated with germline mutations in *PTCH1 or SUFU*, and *TP53*, respectively (6–12). Individuals with Familial Adenomatous Polyposis (FAP) caused by pathogenic variants in *APC* have also been linked to medulloblastoma, sometimes referred to as Turcot Syndrome Type 2 (10).

In addition to *PTCH1*, *SUFU*, *TP53* and *APC*, recent medulloblastoma studies have identified germline pathogenic or likely pathogenic (P/LP) variants in a few well described cancer susceptibility genes (CSG), including *ELP1* and *GPR161* (13–17). Homozygous and compound heterozygous *BRCA2* and *PALB2* P/LP variants have also been identified in a subset of patients with SHH subgroup medulloblastoma with Fanconi Anemia caused by homologous recombination repair deficiency, though the estimated risk of developing medulloblastoma from a single P/LP variant in *BRCA2* or *PALB2* remains low (15, 18). Approximately 5% of all pediatric patients with medulloblastoma have been found to carry a P/LP variant in a CSG. The studies ranged from 120 to 1,022 participants, and the frequency of these variants varied based on subgroup with up to 20% in the SHH subgroup and only 0-2% in the Group 3 and Group 4 subgroups (14–17).

Despite the importance of germline susceptibility in a subset of patients with medulloblastoma, the rates of P/LP variants in CSGs remain uncertain because of the heterogeneity of medulloblastoma and variation in study designs particularly related to genes evaluated, participant demographics, and pathogenicity pipelines. To better understand the genetic etiology of medulloblastoma, we performed a comprehensive exome sequencing analysis of 160 pediatric medulloblastoma survivors and characterized the frequency of germline pathogenic variants in CSGs compared to 1,259 cancer-free controls.

## Methods

### Study population

A total of 160 survivors of medulloblastoma were assembled from two studies, including 134 participants from the Childhood Cancer Survivor Study (CCSS; named Case set 1) and 26 participants diagnosed at the Children’s National Medical Center (CNMC; named Case set 2). The CCSS is a multi-institutional effort funded by the National Cancer Institute (NCI) grant U24CA55727 of the National Institutes of Health since 1994. It established a large cohort of 14,361 participants who survived childhood or adolescent cancer for five or more years diagnosed at one of 26 North American hospitals between 1970 and 1986 (19–21). CCSS participants are younger than 21 years of age at diagnosis and various cancer types were included, with medulloblastoma characterizing 2.6% of participants (13). The participants diagnosed at CNMC are part of a medulloblastoma natural history study conducted at the NCI (22, 23), and all participants with available DNA for germline exome sequencing were included in this study. Controls consisted of 1,259 cancer-free adults (not selected for family history of cancer) from two large studies: the Prostate, Lung, Colon and Ovarian Cancer Prevention clinical trial (n=1,054; mean age of 62.5 years) (24, 25), and the American Cancer Society Cancer Prevention Study II (ACS; n=205; mean age of 62.8 years); hereafter, “controls” (26). Overall, the controls were 50% male and 74% European (**Supplementary Table 1**). The genetic ancestry was determined for the study group and the control group using exome sequencing data based on structure and principal component analyses, as previously described and further details in **Supplemental Methods** (27). Participants with more than 80% European ancestry were considered European. All participants provided written informed consent and were recruited through institutional review board-approved protocols.

### Exome sequencing and variant analysis

Germline DNA was extracted from whole blood or buccal cell samples for all participants. Exome sequencing was performed at the Cancer Genomics Research Laboratory, National Cancer Institute (CGR, NCI) as previously described (13, 28, 29). All participants and controls were jointly called with comparable QC and coverage. In brief, variants were aligned to reference genome hg19 with NovoAlign (http://www.novocraft.com) and then jointly called by FreeBayes (v0.9.14), GATK UnifiedGenotyper (v3.1), and GATK HaplotypeCaller (v3.3). Poor quality and contaminated samples were excluded from the dataset; any variants that were flagged with our custom pipeline quality control metric (CScorefilter), had a read depth < 10, ABHet < 0.2 or > 0.8, or genotype quality scores <20 were excluded from the analysis. The average read depth was 50X in participants and 55X in controls (**Supplementary Figure 1**). The analysis was restricted to variants with a minor allele frequency (MAF) of less than 1% in all non-cancer ethnic subgroups in The Genome Aggregation Database (gnomAD, version 2.1.1, excluding cancer), Exome Aggregation Consortium (ExAC, excluding the Cancer Genome Atlas data) (30), 1000 Genomes Project (31), and the Exome Sequencing Project (32). Please see **Supplemental Methods** for additional information on exome sequencing.

### Cancer-susceptibility genes and pathogenicity classification

We investigated 239 candidate CSGs from published studies (12, 14–17, 33, 34), including six autosomal dominant (AD) genes known to predispose to medulloblastoma (*SUFU, ELP1, GPR161, PTCH1, APC*, and *TP53*). In addition, we evaluated two additional pathways known to be important in medulloblastoma: SHH pathway (16 total genes) and WNT pathway (24 total genes) (35–37). CSGs were grouped by the previously reported mode of inheritance, including autosomal dominant (AD; n=167), autosomal recessive (AR; n=54), X-linked recessive (XLR; n=10), Y-linked recessive (YLR; n=1), and uncertain mode of inheritance (n=7); we additionally created a subcategory of known germline medulloblastoma genes, as noted above, that overlapped with the AD genes (n=6). The total list of candidate genes is summarized in **Supplementary Table 2**, and CSG mode of inheritance was determined by the original publication and confirmed by gene review.

Variants were characterized as pathogenic (P), likely pathogenic (LP), variant of uncertain significance (VUS), likely benign (LB), or benign (B) using a hierarchical classification system based on ClinVar and InterVar as outlined in **Supplementary Figure 2**. Our classification system was based on previous reports (13, 27) and on the guidelines recommended by the American College of Medical Genetics and Genomics and the Association for Molecular Pathology (38). Variants of unknown significance in the six genes known to predispose to medulloblastoma were further characterized as LP if predicted as loss of function (pLoF) by SnpEff (39). All P/LP variants observed in the participants are described in **Supplementary Table 3**. All P/LP variants had their sequence reads (BAM files) manually reviewed using Integrative Genomics Viewer (IGV) to exclude potential sequencing and analysis artifacts that could represent false positives (40).

### Statistical methods

We compared the frequency of P/LP variants in cases to that of 1,259 controls using Fisher’s exact tests. To mitigate type I error from multiple tests, a Bonferroni correction was applied based on the number of genes with a P/LP variant detected (n=30) that were compared among the cases and controls, and a p‐value threshold of <1.6 x 10^-3^ was considered statistically significant. For the purposes of this study, the terms “nominally significant” and “enriched” refer to results with a p-value <0.05 but not meeting the Bonferroni threshold, and “statistically significant” refers to results with a p-value <1.6 x 10^-3^. We also compared differences between cases with and without a P/LP variant by ancestry, sex, vital status, and age at diagnosis, using Fisher’s exact tests and Mann-Whitney U Tests.

## Results

We assessed the frequency of CSG P/LP variants in 160 pediatric survivors of medulloblastoma. 90% of cases (n=144) were of European ancestry, the median age at diagnosis was 7.6 years (age range, 0.5-22.4 years), and 56.3% (90 cases) were male (**Table 1**). Most cases were alive at last follow-up (91%) with the time of follow-up longer for Case set 1 versus Case set 2, and the median length of follow-up time was 32.2 years versus 7.7 years, respectively.

**Table 1:** Demographics and clinical characteristics of all participants.

Characteristics	Case set 1, No. (%)	Case set 2, No. (%)	Total (%)
Total Participants			
	134 (83.8)	26 (16.3)	160 (100)
Sex			
Male	74 (55.2)	16 (61.5)	90 (56.3)
Female	60 (44.8)	10 (38.5)	70 (43.8)
Ancestry			
European	123 (92)	21 (80.8)	144 (90)
Non-European	11 (8)	5 (19.2)	16 (10)
Vital Status			
Alive	120 (89.6)	26 (100)	146 (91.3)
Dead	14 (10.4)	0 (0)	14 (8.8)
Median Diagnosis Age in years (range)			
	7.9 (0.5-18.3)	5.3 (1.8-22.4)	7.6 (0.5-22.4)
Follow Up Duration in years (range)			
	32.2 (12.5-44.4)	7.7 (0.8-19.4)	30.6 (0.8-44.4)

Case set 1 are participants from the Childhood Cancer Survivor Study and Case set 2 are from the natural history study conducted at the National Cancer Institute, diagnosed at Children’s National Medical Center. European defined as CEU >80% using genotyping data (Northern and Western European ancestry). The non-European participants included nine cases of African ancestry and two of Asian ancestry from Case set 1 and three cases of African ancestry and two of Asian ancestry in Case set 2.

**Figure 1:**
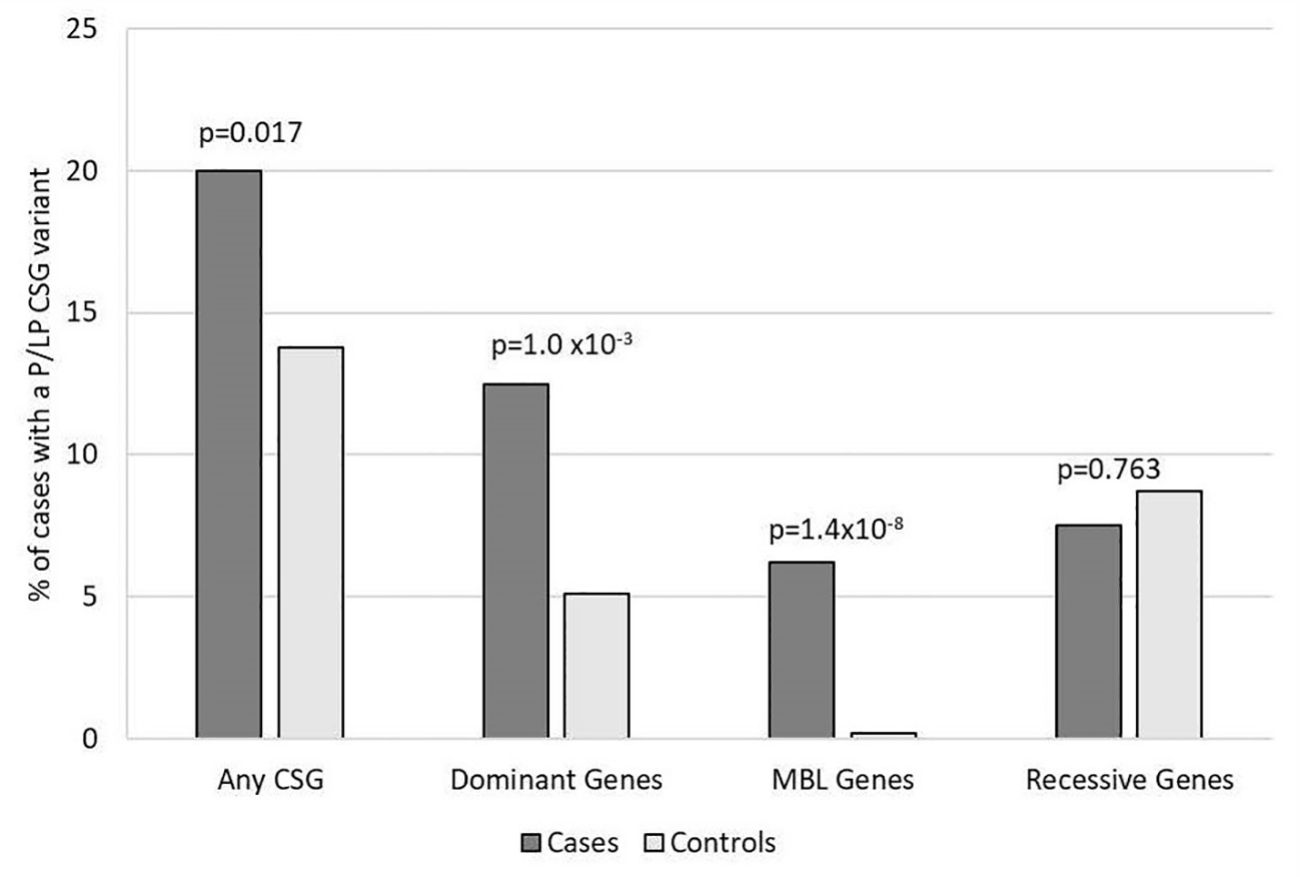
Frequency of rare pathogenic or likely pathogenic variants in cancer susceptibility genes in participants with medulloblastoma vs. controls. Includes all 160 medulloblastoma survivors and 1,259 cancer-free controls. Variants of unknown significance in known medulloblastoma CSGs predicted to be loss of function are included with P/LP variants for autosomal dominant and MBL genes. CSG, cancer susceptibility genes; P, pathogenic; LP, likely pathogenic; MBL genes, six genes known to predispose to medulloblastoma.

Overall, 33 (20%) medulloblastoma cases had a total of 40 germline P/LP variants in at least one of the 239 CSGs, which was nominally higher than the frequency observed in the controls (13.8%, 174/1259; p= 0.017) (**Figure 1**). Restricting the analysis to the European cases and controls, the results showed similar patterns, but were not significantly different; 21% of cases and 14.8% of controls had a P/LP variant (p=0.084).

**Figure 2:**
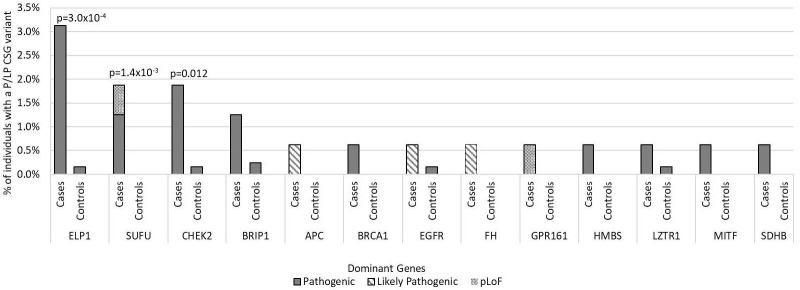
Frequency of pathogenic and likely pathogenic variants in cancer susceptibility genes with autosomal dominant inheritance in medulloblastoma cases vs. controls by gene of interest. Includes all 160 medulloblastoma survivors and 1,259 cancer-free controls. Predicted loss of function (pLoF) included two variants in known medulloblastoma CSGs. Only p-values <0.05 are noted in the figure.

For the 196 AD CSGs and WNT/SHH pathway genes, the frequency of P/LP variants in all cases [12.5% (20/160)] was statistically significantly higher versus 5.1% of controls (65/1259; p=1.0 x10^-3^); the results were similar when restricted to cases of European ancestry (12.4% vs. 5.3%; p=2.5 x10^-3^). 18 of the 20 cases with AD P/LP variants were classified based on ClinVar or InterVar, and two were classified based on pLoF (**Supplementary Table 3**), and none of these same AD P/LP variants were found in the controls. 3.1% (5/160) of cases had more than one P/LP variant, compared to 1.3% of the controls (p=0.078), and these individuals had either two dominant P/LP variants, a dominant and a recessive P/LP variant, or two recessive P/LP variants in different genes. Cases with an AD P/LP variant (n=20) did not differ significantly from cases without (n=140) by age, ancestry (European vs. non-European), sex, or vital status (**Supplementary Table 4**). The AD CSGs with the highest number of P/LP variants in cases, and statistically significantly higher than controls, were *ELP1* (5 total cases, one was non-European ancestry; p=3.0x10^-4^) and *SUFU* (3 European cases; p=1.4x10^-3^). *CHEK2*, which has not been previously associated with medulloblastoma, had nominally more P/LP variants when compared to controls (3 European cases; p=0.012) (**Figure 2**). A pathogenic variant in *BRCA1* was also noted and was the only *BRCA*-associated medulloblastoma in our cohort.

In the known medulloblastoma predisposition AD genes, 6.3% of cases (10/160) had a P/LP variant, which was statistically significantly higher than observed in controls (0.2%, 2/1259; p= 1.4x10^-8^), and none of these same P/LP variants in the cases were also found in the controls. We identified P/LP variants in four of the six medulloblastoma genes: *APC*, *ELP1*, *GPR161*, and *SUFU* (**Supplementary Table 3**). The case-control results were similar when restricted to European ancestry individuals (5.6% of cases vs. 0.1% of controls; p=3.9x10^-7^). The cases with a medulloblastoma gene P/LP variant (n=10) did not significantly differ from cases without (n=150) by age, sex, ancestry, or vital status (**Supplementary Table 4**). We did not observe any P/LP variants in the WNT pathway genes or SHH pathway, except for *SUFU*.

**Figure 3:**
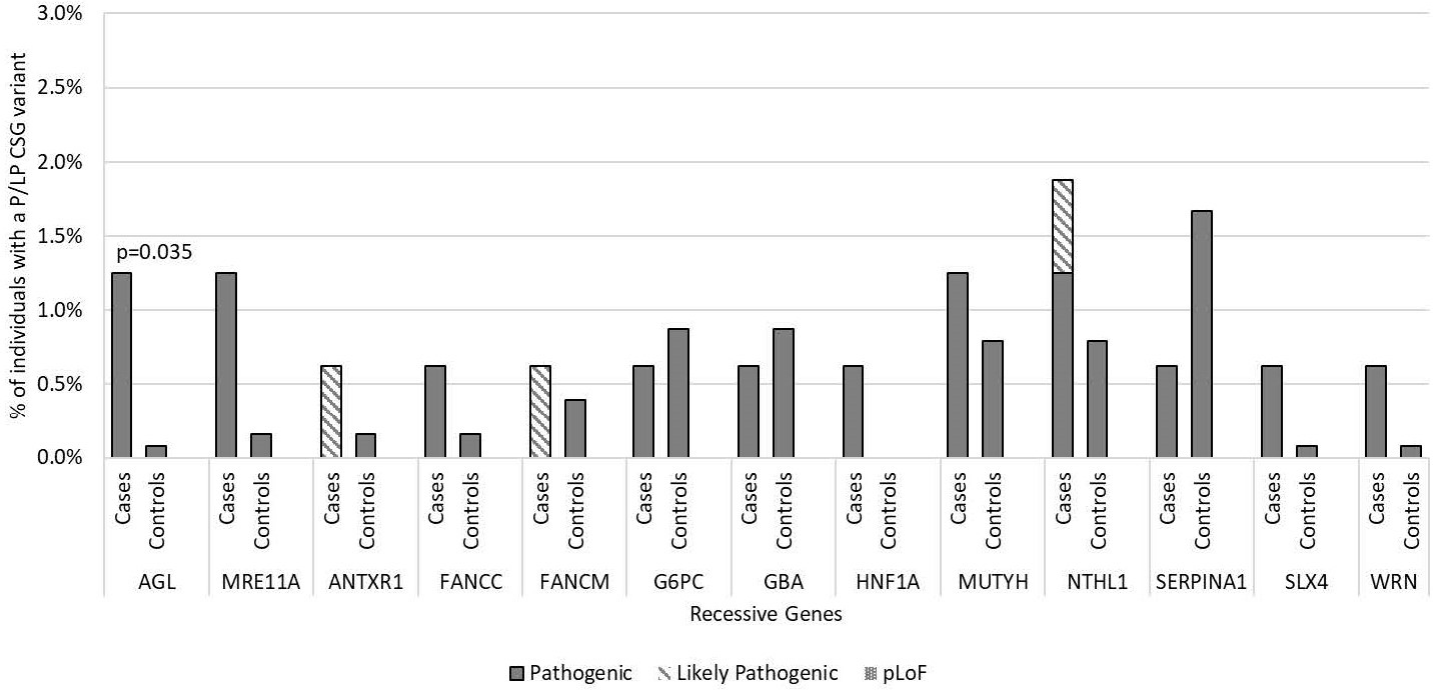
Frequency of pathogenic and likely pathogenic variants in cancer susceptibility genes with autosomal recessive inheritance in medulloblastoma cases vs. controls by gene of interest. Includes all 160 medulloblastoma survivors and 1,259 cancer-free controls. Predicted loss of function (pLoF) included two variants in known medulloblastoma CSGs only. No participants were found with homozygous or compound heterozygous inheritance P/LP variants in these recessive cancer susceptibility genes or to have predicted loss of function. Only p-values <0.05 are noted in the figure.

In the 56 AR CSGs, we did not identify any cases who were homozygous or compound heterozygous carriers of P/LP variants in AR genes. There was also no difference between the carrier frequency of heterozygous P/LP variants in the cases and controls in the AR CSGs; 8.3% of European cases (12/144) had a heterozygous P/LP variant versus 9.5% of European controls (89/937; p=0.76). Furthermore, five AR genes had the same P/LP variants in the cases (*FANCC*, *G6PC*, *MUTYH*, *NTHL1*, *SERPINA1*) and in the controls (**Supplementary Table 3**). *AGL* was the only AR gene that had P/LP variants enriched in cases compared to controls (p=0.035; **Figure 3**) and has not been previously associated with medulloblastoma. No P/LP variants were identified in the ten XLR genes, the single YLR gene, or the 7 genes of unknown inheritance.

We identified multiple P/LP variants in 4.3% of cases (7/160), two included a known medulloblastoma gene, versus 1.3% in controls (16/1259; p=0.014), none of these were in a known medulloblastoma gene.

## Discussion

We identified a statistically significant enrichment of P/LP variants in dominantly inherited CSGs in approximately one eighth of 160 pediatric medulloblastoma survivors compared to 1,259 cancer-free controls with comparable exome data. This was mainly driven by 6.3% of the survivors harboring a P/LP variant in a known AD medulloblastoma gene. The CSGs with the most P/LP variants in cases, and significantly higher than controls, were the known medulloblastoma genes *ELP1* and *SUFU.*

It is difficult to directly compare the frequencies of CSG P/LP variants across studies due to differences in variant classifications, databases used, and the two largest medulloblastoma germline genetic studies to date included both survivors and non-survivors. In a cohort of 230 cases with all subtypes of medulloblastoma represented, Grobner et al. (14) reported that 6% of all cases harbored a P/LP variant in a CSG, compared to our cohort at 12.5% (20/160) using their gene list. In a large cohort of 1,022 patients with medulloblastoma, Waszak et al. (15) reported a P/LP carrier prevalence of 5.6%, compared to 12.5% (20/160) in our cohort when restricting to their gene list. These differences could also be due to different classifications of pathogenicity across the studies and may not represent a true difference in P/LP variant prevalences among survivors and non-survivors. We used ClinVar, InterVar, and predicted LOF variants for our pathogenicity classifications, which were not all incorporated in the other pipelines. The two predicted LOF variants should be interpreted with caution since they have not been previously reported and we do not have functional data to confirm pathogenicity. It is also important to acknowledge that only the P/LP variants in medulloblastoma genes have been previously associated with medulloblastoma. The other P/LP variants should be replicated in additional studies to confirm their pathogenicity. However, 85% of our P/LP variants were identified by ClinVar, and even if we only used ClinVar classifications, 11% of cases carried a P/LP variant in an AD CSG and 4% in a known medulloblastoma gene. Some ClinVar classifications are expected to change over time as knowledge advances, which likely also contributed to differences based on ClinVar information at different times. Similarly, a previous exome study across all pediatric cancer survivors that included many participants in this study had identified 6.3% of medulloblastoma cases with a P/LP variant in an AD CSG (13); this study also used ClinVar classifications and illustrates the dynamic nature of these classifications.

In our study, the CSGs with the highest number of P/LP variants in cases versus controls included *ELP1* and *SUFU.* The prevalence of P/LP variants in *ELP1* in our cases (3.1%, 5/160) was similar to what has previously been reported in a large cohort of cases with medulloblastoma (3.2%, 23/713) (16). In this larger cohort, *ELP1* was associated with a more favorable outcome, with a five-year overall survival rate of 92%. In our study, cases with P/LP variants in *ELP1*, although based on small numbers, seemed to also have favorable outcomes. The median age of diagnosis was 10.3 years (range 3.7-14.6), and four of the five cases were alive at last follow-up, with a median overall survival of 30 years (range 19.4-36.5). *ELP1* is a subunit of the elongator complex and was recently discovered to be pathogenic for SHH medulloblastoma, while other subunits have previously been linked with other cancers such as breast cancer and melanoma (41). The consistent prevalence of P/LP variants in *ELP1* in multiple cohorts provides supportive evidence for adding *ELP1* to cancer susceptibility gene lists, especially for medulloblastoma. The prevalence of P/LP variants in *SUFU* in our survivors (1.9%) also compares similarly to what has been previously reported (15). Patients with medulloblastoma who harbor P/LP variants in *SUFU* typically present in infancy with a median age at diagnosis of 1.5 years (14, 15), and two of our three cases were diagnosed at similar ages (age at diagnosis for these cases were 1 year, 1.5 years, and 4 years).

There were two genes (*CHEK2* and *AGL*) that had P/LP variants enriched in medulloblastoma cases compared to controls that were not previously associated with medulloblastoma. *CHEK2* is a tumor suppressor gene with a large footprint in the literature primarily focused on associations with hereditary nonpolyposis colorectal cancer and breast cancer (42), but given its high mean allele frequency in the general population, it is difficult to interpret its importance as a susceptibility gene here (43). Loss of *AGL* is classically associated with glycogen storage disease and has also been found to be associated with bladder cancer and proliferation of bladder cancer cells, although none of the patients in the current study had bladder cancer (44, 45). *CHEK2* and *AGL* require follow-up in future studies to determine their relationship with susceptibility to medulloblastoma.

Two notable genes not observed to have any P/LP variants in our cohort were *PTCH1* and *TP53*. Cases from our smaller cohort from CNMC (Case set 2) with NBCCS that had variants in *PTCH1* were excluded from the current study (22). Interestingly, the larger group of cases from the CCSS cohort (Case set 1) had no variants in *PTCH1*. In previous larger studies, the prevalence of *PTCH1* ranged from 0.4% (14) to 4.5% (17). P/LP variants in *PTCH1* have been associated with infant medulloblastoma (14), and since our cohort has a limited number of infants this may be a factor. P/LP variants in *TP53* are associated with both poor medulloblastoma survival and subsequent primary cancers, and thus are less likely to be identified in a long-term survivor cohort. A P/LP variant in *GPR161* was present in only one case. Similar to *SUFU*, *GPR161* variants seem to be found primarily in infant medulloblastoma; previously reported in 3.4% of pediatric SHH medulloblastoma cases (5 of 6 of our reported cases were under the age of 12 months) (17), compared to only one 10-year-old case with a P/LP *GPR161* variant in our cohort. Of note, only six cases from our study were diagnosed at less than one year of age.

A unique aspect of our study is its focus entirely on a survivor cohort, allowing identification of genes that could be related to prognosis and treatment response, and this likely limited P/LP variants in genes related to poor survival. However, a limitation of our study is that it includes decades-old cases, which limits generalizability to contemporary survivor cohorts, and limits the ability to classify these cases based on the contemporary criteria of different subtypes. In addition, other limitations of our study include the low power for evaluations of individual susceptibility genes, the inability to assess family history in most of our cases, and the potential for selection bias favoring cases with better prognosis that is innate to any survivor cohort.

In summary, one in eight pediatric medulloblastoma survivors in this study had an autosomal dominant germline pathogenic variant in a CSG. As well as confirming results for several known medulloblastoma susceptibility genes, we identified new genes of potential interest in the development of medulloblastoma. Further studies are needed to validate these observations and identify associations by histologic or molecular subtype. Our findings improve the understanding of the germline genetic etiology of pediatric medulloblastoma and provide insight into specifically medulloblastoma survivors. These results could have important implications for genetic testing in pediatric patients and their families with medulloblastoma specifically for known medulloblastoma genes.

## Data availability statement

The datasets presented in this study can be found in online repositories. The names of the repository/repositories and accession number(s) can be found below: https://www.ncbi.nlm.nih.gov/gap/, phs001327.v2.p1.

## Ethics statement

The studies involving humans were approved by the institutional ethics committees of the participating centers and written informed consent was obtained from all individuals. All data received from CCSS were de-identified. The studies were conducted in accordance with the local legislation and institutional requirements. Written informed consent for participation in this study was provided by the participants’ legal guardians/next of kin. Written informed consent was obtained from the individual(s) for the publication of any potentially identifiable images or data included in this article.

## Author contributions

DR: Methodology, Investigation, Formal Analysis, Data curation, Writing – review & editing, Writing – original draft. DG: Methodology, Investigation, Formal Analysis, Data curation, Writing – review & editing, Writing – original draft. JK: Data curation, Writing – review & editing. TS: Resources, Writing – review & editing. GR: Resources, Writing – review & editing. YS: Resources, Writing – review & editing. MG: Writing – review & editing. LM: Resources, Writing – review & editing. MH: Resources, Writing – review & editing. GA: Resources, Writing – review & editing. NF: Resources, Writing – review & editing. WH: Resources, Writing – review & editing. WD: Resources, Writing – review & editing. AL: Resources, Writing – review & editing. WL: Data curation, Writing – review & editing. BH: Data curation, Writing – review & editing. JL: Data curation, Writing – review & editing. AH: Data curation, Writing – review & editing. AG: Methodology, Investigation, Funding acquisition, Formal Analysis, Conceptualization, Writing – review & editing, Writing – original draft, Supervision. LM: Methodology, Investigation, Funding acquisition, Formal Analysis, Conceptualization, Writing – review & editing, Writing – original draft, Supervision.
