# Supplementary material for: Frequency of pathogenic germline variants in pediatric medulloblastoma survivors
Source: Front Oncol. 2024 Aug 9;14:1441958. doi: 10.3389/fonc.2024.1441958 (PMC11341988; doi:10.3389/fonc.2024.1441958)
Supplement: Supplementary file 1 [file DataSheet_1.pdf]

Supplemental Methods:

Exome sequencing was performed at the NCI Cancer Genomic Research Laboratory as previously described (1, 2). Cases and controls were jointly called with comparable QC and coverage. In brief, NimbleGen's SeqCap EZ Human Exome Library, Exome+UTR (Roche NimbleGen, Inc., Madison, WI, USA), capture kit was used for all cases and controls, and sequencing was performed on an Illumina HiSeq2500 (with 125bp paired end reads) with the Bio Nextflex (Perkin Elmer, Inc., Austin, TX) library prep or on an Illumina HiSeq4000 (with 150bp paired end reads) with Kapa HyperPlus (Roche Sequencing and Life Science, Kapa Biosystems, Wilmington, MA) library prep. Samples were then aligned to human genome assembly hg19 with NovoAlign (http://www.novocraft.com) and variants were jointly called using GATK HaplotypeCaller (v3.3), GATK UnifiedGenotyper (v3.1), and FreeBayes (v0.9.14). Multiallelic variants were split and left-aligned and trimmed prior to integrating the variant information from the three callers using ArmyKnife (v2.2.36). Variants were retained if called by two of three callers. Individual genotypes were retained if the HaplotypeCaller genotype quality (GQ) score was ≥20 and an alternative allele read depth >10. Genotypes were removed for intermediate quality samples if the hetAB < 0.2 or >0.8. The average depth was 50X in cases and 55X in controls (Supplemental Figure 1). Exome sequenced data has been previously published (1, 2), and publicly available at dbGAP accession: phs002072.v1.p1. Subject ancestry was estimated using LASER (3) by analyzing the short read sequences and procrustes against 938 HDGP reference samples. All variants from the ClinVar database (as of 9/8/2023) had to meet the minimum requirements for data sharing to support quality assurance ("badged lab") by ClinGen (https://www.clinicalgenome.org/lablist/); and, all of the "non-badged lab" calls were disregarded (4). If classifications were conflicting, it was resolved by majority rule.

References:

1. Kim J, Gianferante M, Karyadi DM, Hartley SW, Frone MN, Luo W, et al. Frequency of Pathogenic Germline Variants in Cancer-Susceptibility Genes in the Childhood Cancer Survivor Study. JNCI Cancer Spectrum. 2021;5(2).
2. Morton LM, Karyadi DM, Hartley SW, Frone MN, Sampson JN, Howell RM, et al. Subsequent Neoplasm Risk Associated With Rare Variants in DNA Damage Response and Clinical Radiation Sensitivity Syndrome Genes in the Childhood Cancer Survivor Study. JCO Precis Oncol. 2020;4.
3. Wang C, Zhan X, Liang L, Abecasis GR, Lin X. Improved ancestry estimation for both genotyping and sequencing data using projection procrustes analysis and genotype imputation. Am J Hum Genet. 2015;96(6):926-37.
4. Landrum MJ, Lee JM, Benson M, Brown GR, Chao C, Chitipiralla S, et al. ClinVar: improving access to variant interpretations and supporting evidence. Nucleic Acids Res. 2018;46(D1):D1062-d7.
